# Innate-Like Lymphocytes Are Immediate Participants in the Hyper-Acute Immune Response to Trauma and Hemorrhagic Shock

**DOI:** 10.3389/fimmu.2019.01501

**Published:** 2019-07-11

**Authors:** Joanna Manson, Rosemary Hoffman, Shuhua Chen, Mostafa H. Ramadan, Timothy R. Billiar

**Affiliations:** ^1^Department of Surgery, F1281 Presbyterian University Hospital, University of Pittsburgh, Pittsburgh, PA, United States; ^2^Barts Centre for Trauma Sciences, Blizard Institute, Queen Mary University of London, London, United Kingdom

**Keywords:** trauma, major trauma, immune response, MODS, innate immunity, innate-like lymphocytes, trauma immunology

## Abstract

Adverse outcomes following severe traumatic injury are frequently attributed to a state of immunological dysfunction acquired during treatment and recovery. Recent genomic evidence however, suggests that the trajectory toward development of multiple organ dysfunction syndrome (MODS) is already in play at admission (<2 h following injury). Improved understanding of the molecular events during the hyper-acute immunological response to trauma, <2 h following injury, may reveal opportunities to ameliorate organ injury and expedite recovery. Lymphocytes have not previously been considered key participants in this early response; however, two observations in human trauma patients namely, raised populations of circulating NKT and NK cells during the hyper-acute phase and persistent lymphopenia beyond 48 h show association with the development of MODS during recovery. These highlight the need for greater understanding of lymphocyte function in the hyper-acute phase of inflammation. An exploratory study was therefore conducted in a well-established murine model of trauma and hemorrhagic shock (T&HS) to investigate (1) the development of lymphopenia in the murine model and (2) the phenotypic and functional changes of three innate-like lymphocyte subsets, NK1.1+ CD3–, NK1.1+ CD3+, γδTCR+ CD3+ cells, focusing on the first 6 h following injury. Rapid changes in phenotype and function were demonstrated in these cells within blood and spleen, but responses in lung tissue lagged behind. This study describes the immediacy of the innate-like lymphocyte response to trauma in different body compartments and considers new lines for further investigation to develop our understanding of MODS pathogenesis.

## Introduction

Severe traumatic injury activates an acute inflammatory response, which increases in extent and magnitude during the first 24 h after injury (1, 2). High levels of inflammatory mediators at admission, are associated with the development of infection, Multiple Organ Dysfunction Syndrome (MODS) and death during hospital admission (1–4). The incidence of MODS is ~30% after severe injury and while advances in trauma resuscitation strategies have reduced early mortality from hemorrhagic shock, they have also likely increased the complexity of survivors (5–7). Organ support is the mainstay of MODS management, resulting in long critical care admissions, late in-hospital deaths and long-term ill-health in some survivors (6, 8, 9).

These adverse outcomes after trauma have, to date, been attributed to an acquired immunological dysfunction which develops during recovery, recent evidence has however demonstrated that the trajectory toward MODS development is already in-play at admission (1, 5, 10, 11). Patients who go on to develop MODS have high numbers of circulating Natural Killer T (NKT) cells and Natural Killer (NK) cells during the hyper-acute phase suggesting that early immune cell mobilization may influence recovery (10–12). Profound lymphopenia then occurs between 4 and 12 h post injury (12). Historically this has been attributed to widespread lymphocyte apoptosis, although the evidence for this is not strong (13, 14). If persistent after 48 h, lymphopenia is associated with MODS development and increased in-hospital mortality (11, 15). These data suggest that lymphocytes, particularly innate-like lymphocytes, play a significant role in the immunological events immediately following injury; much earlier than previously thought. They also indicate that therapeutic intervention in the pre-hospital phase may abrogate organ injury, if we can improve our understanding of the molecular mechanisms involved (16). Although studies to date have focused on peripheral blood because it is an accessible sample medium, cellular activity in other immunological compartments, such as spleen, bone marrow, liver, and lymph nodes may well differ from those in blood. The events in other compartments and the influence of cell mobilization have not yet been considered in clinical studies.

This exploratory study was conducted using a well-established murine trauma and hemorrhagic shock (T&HS) model. The study focused on NK cells, NK-T cells, and δγ T cells. All are lymphocytes with innate-like properties and we hypothesized that they would be active during the hyper-acute immune response to trauma, <2 h following injury (10). Unlike conventional lymphocytes, they can be directly activated by Danger-Associated-Molecular Patterns (DAMPs), cytokines and adrenergic signals to generate acute inflammation (17–22). They also share some common receptors, interact with one another and are known to orchestrate immune responses in other disease settings (17, 23–28). Our first aim was to determine whether peripheral blood lymphopenia occurred in the murine model and at what time point. We hypothesized that this lymphopenia may not be due to whole body lymphocyte loss from apoptosis, but transient redistribution and so sought evidence of cell death and sequestration into other organs. Our second aim was then to examine NK, NKT, and γδ T cells in blood, bone marrow, spleen and lung to assess cell phenotype and activity status, within these different compartments during the first 24 h following injury.

## Materials and Methods

This study was conducted at the University of Pittsburgh in accordance with the National Institute of Health guidelines and with the ethical approval of the University of Pittsburgh, Animal Care and Use committee.

### Subjects

Male, wild-type C57BL/6 mice aged between 8 and 12 weeks and weighing between 20 and 30 g, were obtained from Charles Rivers Laboratories International (Wilmington, Mass). All were housed in pathogen-free conditions with 12 h light-dark cycles and unlimited access to food and water.

### Model Protocol

The tissue trauma and hemorrhagic shock model (T&HS) was performed by experienced operating technicians. The method has been well described in previous literature but shall be detailed in brief (29, 30). A solution of crushed bone fragments was prepared, under sterile conditions, from bilateral femur and tibia harvested from a litter-matched donor. The solution was re-suspended in sterile PBS to achieve a volume of 2 ml. After administration of anesthesia with 70 mg/kg intra-peritoneal (i.p) pentobarbital sodium (Hospira, Inc., Lake Forest, IL, for OVATION Pharmaceuticals, Deerfield, IL), limbs were fixed and bilateral cannulation of femoral arteries performed, via groin incisions, to allow placement of PE-10 catheters. One catheter allowed for real-time BP monitoring (Digi-med BPA 400 Analyzer, Micro-Med Inc, Louisville, KY) and the other enabled blood draw. A bilateral thigh crush was performed using a hemostat for 30 s to induce soft tissue injury, then 0.15 ml of bone solution was injected, via an 18 gauge needle, into each thigh to mimic femur fracture (Pseudofracture, PF). Hemorrhagic shock was induced by withdrawal of blood from the femoral artery cannula to achieve a MAP of 25 mmHg (0 h) which was sustained for 2 h. Time 0 h was defined as the end of the blood draw and the beginning of the shock phase. Animals were resuscitated with Ringer's Lactate solution, at 2 h, using three times the volume of shed blood and given subcutaneous buprenorphine analgesia (0.10 mg/kg, Bedford Laboratories, Bedford, OH). Throughout the experiment, body temperature was maintained by use of a warming plate and controlled ambient room conditions. All subjects had unrestricted access to food and water. Mice were euthanized at 2, 6, or 24 h from the beginning of the hemorrhage induction using excessive isoflurane inhalation (Hospira, Lake Forest, IL, USA). Subjects that were euthanized at the 2 h timepoint did not receive crystalloid resuscitation. Rapid thoracotomy and cardiac puncture were then performed for blood draw in to a heparinized syringe. Intra-cardiac infusion of sterile PBS was performed for systemic vascular flush and then spleen, bone marrow, and lung were harvested.

### Preparation of Single Cell Suspensions

For flow cytometry experiments, single cell suspensions of each organ were prepared in accordance with standard protocols. All centrifuge steps are at 350 g for 5 min at 4°C unless otherwise stated.

#### Blood

Following manual hemocytometer count, blood drawn from the cardiac puncture was centrifuged at 3,400 rpm for 10 min at 4°C, serum was extracted in aliquots and stored at −80°C. One thousand microliter of warmed (37°C) red cell lysis buffer (prepared in-house: 8.26 g NH4Cl, 1 g KHCO3, and 0.037 g ETDA/L) was added to the eppendorf and the liquid aspirated up and down to loosen the remaining cell pellet. This solution was transferred into a 50 ml Falcon Tube using more lysis buffer to swill out remaining cells. Ten milliliter of warm red cell lysis buffer was then added and the solution incubated for 3 min with periodic vortex. Dilution with 20 ml of cold PBS was followed by centrifuge, removal of supernatant and re-suspension of pellet in 2 ml of PBS. The 2 ml solution was transferred to a flow-cytometry tube, centrifuged and resuspended in 200 μl PBS, ready for antibody application.

#### Spleen

The harvested spleen was placed in a Petri dish with DMEM (Dulbecco modified Eagle Media) and mechanically fragmented and filtered (70 μm). This solution was centrifuged, decanted and the pellet re-suspended in 5 ml of red cell lysis buffer, vortexed and diluted to 20 ml with cold PBS. The solution was centrifuged, decanted, and re-suspended in 10 ml of DMEM. Manual hemocytometer count was performed.

#### Bone Marrow

Femur and tibia from both back legs were harvested, cleaned and marrow placed in to a petri-dish. Marrow was fragmented, filtered (70 μm), centrifuged and the pellet re-suspended in 200 μl PBS. Five ml of warmed red cell lysis buffer was added and the solution incubated for 3 min, with periodic vortex. Dilution with 20 ml of cold PBS was followed by centrifuge, supernatant decant and re-suspension in 5 ml of DMEM. Manual hemocytometer count was performed.

#### Lung

A single cell suspension was produced using a Lung dissociation kit (Miltenyi Biotech 130-095-97). A single lung and the enzyme mixture were placed into the gentle MACS C tube and fragmented using Dissociation program 1 (gentleMACS Dissociator, Miltenyi Biotec #130-093-235). The solution was incubated for 30 min at 37°C, followed by a second dissociation programme and centrifuge. The pellet was re-suspended and the solution filtered through a 70 μm sterile filter. Making the volume up to 5 ml with PBS the solution was centrifuged, supernatant decanted and the pellet re-suspended in 5 ml of DMEM. This was over-laid with 5 ml of Lympholyte-M and centrifuged at 1,500 g for 20 min at room temperature, without the brake. The interphase was then aspirated, washed with PBS and centrifuged. Finally, the solution was centrifuged, the supernatant decanted and the final pellet re-suspended in 1 ml of PBS ready for application of antibodies. Manual hemocytometer count was performed.

### Inflammation and Organ Dysfunction

The magnitude of the inflammatory response and end organ damage was quantified using ALT, ALP, and IL-6. These molecules were measured in serum samples drawn at 6 h, which represents the standard end-point for this model. IL-6 was quantified using a commercially available ELISA kit (R&D Systems Inc., Minneapolis, MN, USA). ALP and ALT were measured using a Dri-Chem 7000 Chemistry Analyzer (Heska Co., Loveland, CO, USA). All were measured in duplicate and average values used for analysis. These end-points were used to ensure comparability between experiments.

### Hemocytometer Counts

Standard full blood counts (FBC) were obtained using whole blood and an automated hemocytometer. For flow cytometry experiments leucocyte counts were obtained using a manual hemocytometer. Counts from multiple experiments, were grouped to obtain an average lymphocyte count for each stage (*n* = 5–30). Percentages obtained from the flow cytometry were then multiplied by the average cell count, to calculate absolute cell numbers for data representation and analysis.

### Flow Cytometry

**Figure 1:**
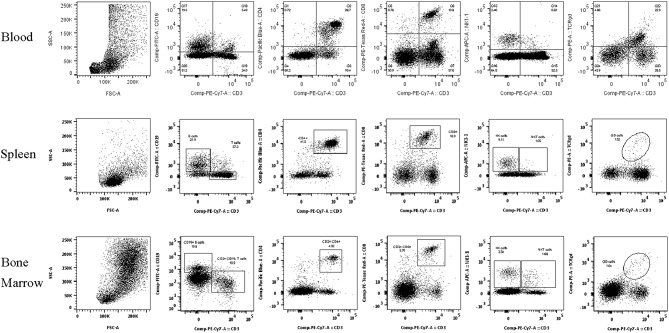
Lymphocyte subset flow cytometry gating strategies. Representative flow cytometry plots demonstrating the gating strategy for the lymphocyte subset identification after removal of doublets and selection of lymphocyte population in **(A)** Blood, **(B)** Spleen, and **(C)** Bone Marrow.

**Figure 2:**
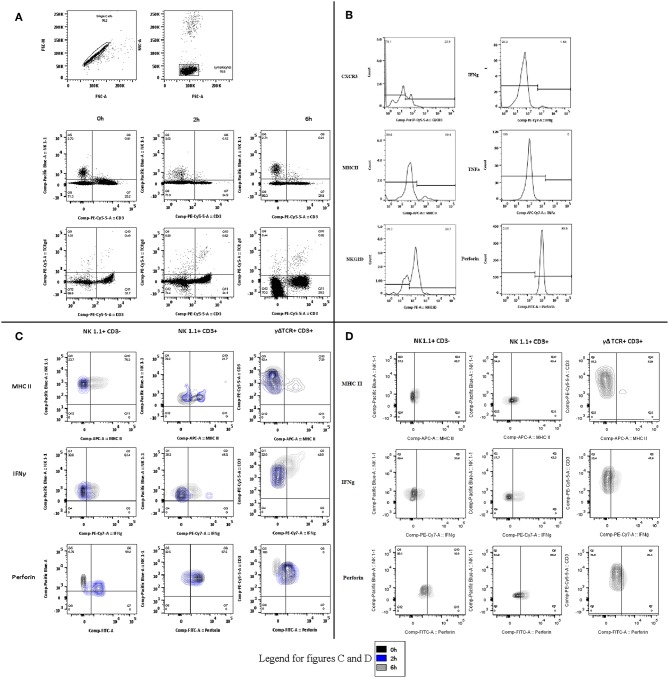
Phenotype and function flow cytometry gating strategies **(A)** Gating strategies for phenotype and function experiments in blood, spleen, and lung. Following identification of single cells, the lymphocyte population was selected and divided by CD3 and either NK1.1 or TCRγδ. Representative plots are displayed for 0, 2, and 6 h. **(B)** Phenotype and function. The phenotype markers (CXCR3, MHCII, and NKG2D) and functional markers (Intracellular IFNγ, TNFα, and perforin) were then examined and recorded as percentage of positive cells for each lymphocyte subset. Representative histograms are displayed. **(C)** Changes in blood over time. Contours plots were overlaid to demonstrate the shifts in phenotype and function within each lymphocyte subset at the different sample points in the protocol. Representative plots of NK1.1 CD3–, NK1.1 CD3+, and CD3 TCRγδ are displayed for blood-borne cells at 0, 2, and 6 h. **(D)** Lung lymphocytes. As in **(C)**, the same process was followed to demonstrate the changes by cell type over time, in this case only Control and 6 h.

Using ~5 million cells per tube, an antibody “master mix” was then added to each tube. The exception was the peripheral blood which had a much lower cell yield (0.5–1.5 million). Concentrations of antibodies were pre-titrated (Box 1). The eBioscience staining protocols were used for cell surface and intracellular antibody staining. A viability marker was used in all experiments. Samples were analyzed using the BD LSR Fortessa (BD Biosciences). The innate-like cells were defined as follows: NK cells = CD3– NK1.1+, NKT cells = CD3+ NK1.1+, γδ T cells = CD3+ γδTCR+. The other leucocyte sub-groups were defined as: CD3+ CD4+, CD3+ CD8+, CD3– CD19+, and CD3– Gr-1+. The phenotypic markers selected were, CXCR3 a marker of migration potential, MHCII a marker acquired following activation or DC cross-talk (16, 22) and NKG2D an activation marker. The intracellular cytokines were IFNγ, TNFα, and perforin. The apoptosis assay was conducted using a commercially available kit (#559763 BD Biosciences) and analyzed using an LSR Cytometer (Figures 1, 2).

Box 1 **Antibodies used for flow panels.**

**Table:** 

Viability Dye AmCyan (#65-0866-14) Fc Blocker (#553141) CD45 APC-Cy7 (#47-0112-82) CD3 PE-Cy5.5 (#35-0031-82) PE-Cy7 (# 25-5711-82) PE (# 12-0033-82) CD4 e450 (#48-0042-82) CD8 PE Tex Red (# MCD0817) CD19 FITC (#11-0191-82) NK1.1 eF450(#MM6628)	(NK1.1 APC # 17-5941-82) Gr-1 AF700 (#56-5931-82) CD183 (CXCR3) PerCP-Cy5.5 (#45-1831-82) MHCII APC (#17-5320-82) CD314 (NKG2D) PE (# 12-5882-82) TCR*γδ* PE-Cy5 (#15-5711-82) IFNγ PE-Cy7 (#25-7311-82) TNFα APC-Cy7 (#502944 Biolegend) Perforin FITC (#11-9392-82)

All antibodies were obtained from eBioscience except Fc Blocker (BD Biosciences) and TNFα (Biolegend)

### Statistics and Data Analysis

All data were collected in Excel (Microsoft, USA) and analyzed using Excel or Graph Pad (Prism, USA). The flow cytometry data was analyzed using FlowJo (Treestar, OR, USA). Flow cytometry data are graphically presented as median (min-max) and quoted as median (IQR). These were tested with Kruskal Wallis and Dunns multiple comparison test. All other values are presented as mean (standard error of the mean: SEM) and analyzed using parametric statistics. The statistical applications are detailed in the results.

## Results

### At 6 h Following T&HS Lymphocyte Count Was Reduced in Blood and Lung, Static in Spleen and Increased in Bone Marrow

**Figure 3:**
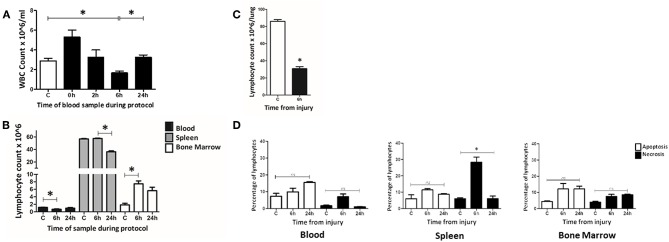
At 6 h following T&HS lymphocyte count was reduced in blood and lung but static or increased in the lymphoid tissues. **(A)** Peripheral blood leucocyte count was determined manually on samples taken at intervals through the T&HS protocol. An initial leukocytosis at the onset of hemorrhagic shock was followed by leucopenia at 6 h. This returned to baseline by 24 h. *C* = 2.9 (2.6–3.1), 0 h = 5.3 (4.6–6.0), 2 h = 3.3 (2.5–4.0), 6 h = 1.7 (1.5–1.8), 24 h = 3.3 (3.0–3.5). Data are presented as mean (SEM) count × 10^6^/ml and tested using Kruskal Walis and Dunn's multiple comparison post-test. **(B)** The lymphocyte population in blood, spleen and bone marrow was quantified, at intervals through the protocol, using flow cytometry. The lymphopenia demonstrated in blood at 6 h was not mirrored in the other lymphoid organ compartments. In spleen, lymphocyte count was static at 6 h but reduced by 24 h. In bone marrow, the lymphocyte count had increased by 6 h and remained above baseline at 24 h. Control(C) denotes a naïve subject of the same strain and age. C, 6 h, 24 h = Blood: 1.2 (0.03), 0.7 (0.11), 1.0 (0.18). Spleen: 56.7 (0.71), 57.5 (0.57), 36.3 (1.36). Bone marrow: 1.9 (0.43), 7.5 (0.77), 5.6 (0.93). Data are presented as mean (SEM) absolute cell count × 10^6^/ml of blood/spleen/both hind limbs (*n* = 6–19). Data were tested using Kruskal Walis with Dunn's multiple comparisons test. All had a *p-*value < 0.01 and *denotes the positive comparisons. **(C)** The lymphocyte count in lung was examined at 6 h using flow cytometry. Lung was selected as a representative, non-lymphoid organ. By 6 h, the absolute count of lymphocytes in the lung tissue had reduced almost threefold C = 86 (84–87), 6 h = 31 (28–32), *p* < 0.01 (*n* = 6). Data are presented as mean (SEM) and tested with Mann Whitney *U* test, *denotes *p* < 0.05. **(D)** Evidence of widespread lymphocyte apoptosis was sought using a cell death assay on samples of blood, spleen, and bone marrow taken at 6 and 24 h following T&HS. Apoptosis was defined as Annexin V + 7AAD- and necrosis was defined as Annexin V+ 7AAD+. An increase in necrosis was observed in the spleen at 6 h. In all other organs and timepoints, there were no significant changes in apoptosis or cell death over the 24 h protocol. Blood: Apoptosis = 6 (6–9), 7 (7–10), 16 (15–16), *p* = 0.15. Necrosis = 2 (2–2), 7 (4–10), 1 (1–1), *p* = 0.08. Spleen: Apoptosis = 4 (4–7), 12 (10–13), 9 (9–9), *p* = 0.10, Necrosis = 5 (4–6), 29 (10–32), 6 (5–7), *p* = 0.02. Bone Marrow: Apoptosis = 4 (4–5), 12 (9–15), 12 (11–13), *p* = 0.10, Necrosis = 4 (3–5), 7 (6–8), 9 (8–9), *p* = 0.06. C denotes a naïve subject of the same strain and age. Data are presented as median (IQR) and tested with Kruskal Wallis and Dunn's comparison of all columns, *denotes *p* < 0.05.

A manual hemocytometer count of peripheral blood leucocytes was conducted to determine changes at set intervals during a 24 h murine trauma and hemorrhagic shock (T&HS) protocol (Figure 3A). At the beginning of the hemorrhagic shock period (0 h) a leucocytosis was observed. This became a leucopenia by 6 h and returned to baseline by 24 h. Flow cytometry was then used to examine changes in lymphocyte count in several different tissue compartments, namely blood, spleen and bone marrow. A significant drop in lymphocyte count was demonstrated at 6 h in peripheral blood but this was not mirrored in spleen or bone marrow (Figure 3B). The lymphocyte count in the spleen remained static at 6 h but fell by 24 h. In bone marrow, the lymphocyte count had increased at 6 h and remained elevated at 24 h. An automated full blood count revealed a fall in hemoglobin at 6 and 24 h, as would be expected of this model [*C* = 13.3(12.1–14.5), 6 h = 7.6(6.8–8.3), 24 h = 6.2(5.8–6.6) × 10^3^/ul, *p* < 0.01 C vs. 24 h].

To investigate this sudden loss of lymphocytes from circulation, evidence of migration into other “non-lymphoid” tissues was sought. We chose to examine lung as it is commonly involved in human MODS. Lung tissue was examined in Control and 6 h samples using flow cytometry (Figure 3C). A marked decrease in the percentage of lymphocytes within lung tissue was observed at 6 h [*C* = 66% (65–68), 6 h = 39% (36–41), *p* = 0.02]. This was not unexpected as there is a known influx of granulocytes at this timepoint; however, when calculated, the lymphocyte count also demonstrated an almost 3-fold reduction [*C* = 86 (84–87), 6 h = 31 (28–32), *p* < 0.01]. Data are presented as mean (SEM) and tested with a Mann Whitney *U*-test (31).

To determine whether lymphocyte apoptosis could explain the reduction in circulating lymphocytes, a standard cell death assay was used to examine blood, spleen and bone marrow at 6 and 24 h (Figure 3D). No organ compartment demonstrated a greater percentage of apoptotic cells (Annexin V + 7AAD-) than the Control; however, an increase in lytic lymphocyte cell death (Annexin V+ 7AAD+) was demonstrated in the spleen at the 6 h timepoint [Annexin V+7AAD+: C = 6 (6–7), 6 h = 31 (29–32), 24 h = 6 (5–7), *p* = 0.02]. Data are reported as median (IQR) and tested with Kruskal Wallis and Dunns comparison of all columns. Lymphocyte loss from circulation was demonstrated at 6 h post injury, although this could not be attributed to widespread apoptosis. Lymphocyte loss from the lungs was also observed. In contrast lymphocyte numbers at 6 h were static in the spleen and increased bone marrow.

### T&HS Provokes Dynamic Changes in Lymphocyte Subsets Which Are Cell Specific, Compartment Specific, and Time Dependent

**Figure 4:**
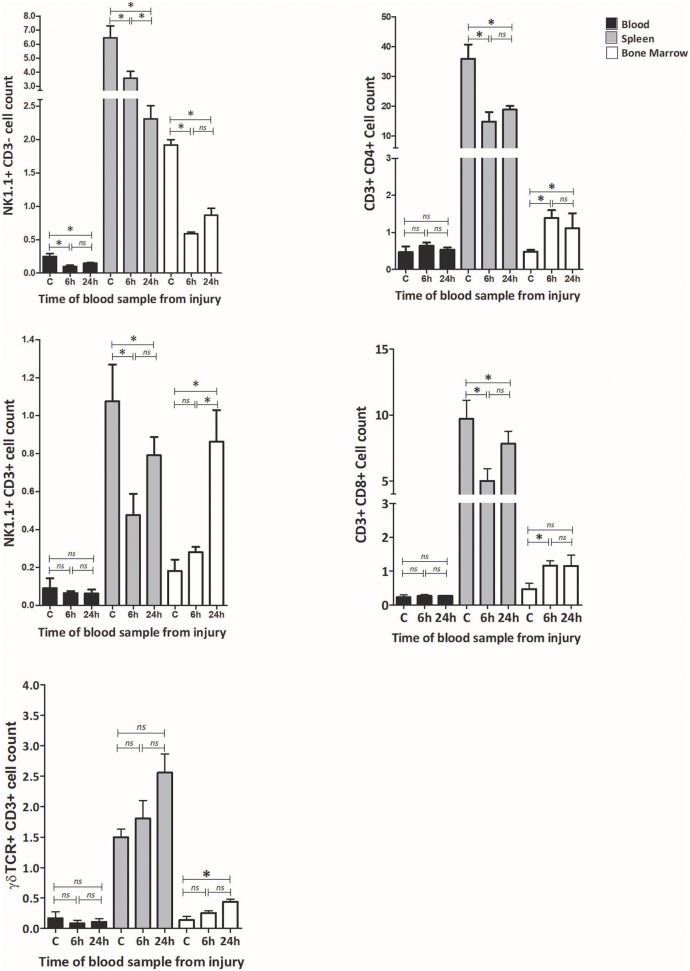
T&HS provokes dynamic changes in lymphocyte subsets which are cell specific, compartment specific and time dependent. Flow cytometry was used to examine the lymphocyte subset populations at 6 and 24 h following injury. Blood, spleen and bone marrow were the compartments selected for examination. Changes in peripheral blood populations of NK 1.1+ CD3– cells were observed at 6 h, while NK1.1+ CD3+, γδTCR+ CD3+ cells, CD3+ CD4+, and CD3+ CD8+ cell populations remained unchanged. In spleen, a decrease in the population of NK 1.1+ CD3–, NK1.1+ CD3+, CD3+ CD4+, and CD3+ CD8+ was observed by 6 h, while γδTCR+ CD3+ cells remained broadly unchanged. In bone marrow, the NK 1.1+ CD3– cell population fell by 6 h while the other cell populations were seen to increase. Examination of lymphocyte sub-set populations revealed dynamic shifts within the immune compartments, principally within the first 6 h. Control (C) denotes a naïve subject of the same strain and age. Data are presented as mean (SEM) absolute cell counts × 10^6^ (per ml of blood, per spleen or per both hind limbs) and tested using Kruskal Wallis with Dunn's multiple comparison of columns, *denotes *p* < 0.05.

Flow cytometry was used to examine changes in the frequency of lymphocyte subsets. The innate lymphocyte subsets including NK cells (NK1.1 + CD3– cells), NKT cells (NK1.1 + CD3+ cells), and γδTCR cells (γδTCR + CD3 + cells) were examined in three immunological compartments; blood, spleen and bone marrow, at 6 h and 24 h post T&HS. Classical T cell subsets, including CD3+ CD4+ cells and CD3+ CD8+ cells were quantified to provide a comparison (Figure 4; Supplementary Table 1).

Within peripheral blood, the population of NK1.1+ CD3– cells fell by 6 h, while all the other populations remained static. Within spleen, a reduction in cell count was observed at 6 h in NK1.1 + CD3–, NK1.1+ CD3+, CD3+ CD4+ and CD3+ CD8+ cells, while γδTCR+ CD3+ cells remained static. In bone marrow, the NK1.1+ CD3– cell population fell dramatically by 6 h. In contrast, CD3+ CD4+ and CD3+ CD8+ cells increased in cell count while NK1.1+ CD3+ and γδTCR+ CD3+ remained static. At 6 h following injury, the NK1.1+ CD3– cell population had reduced in all three of the examined compartments, CD3+ CD4+ and CD3+ CD8+ cells were down in spleen and up in bone marrow. CD3+ γδTCR+ cells increased in bone marrow by 24 h but displayed no earlier changes. Fluctuations in cell populations at 6 h and 24 h following injury were cell specific, tissue specific, and time dependent.

### Innate-Like Cells Change Their Phenotype and Function During the First 6 h Following T & HS

The secondary objective of this study was to investigate changes in phenotype and activity of the innate like cells. As the greatest change in cell numbers occurred within 6 h of injury, we increased our early sampling time-points. Using flow cytometry, the surface markers and intracellular cytokine levels were examined in NK1.1+ CD3–, NK1.1+ CD3+, and γδTCR+ CD3+ cells at 0 h, 2 h, and 6 h following T&HS. Blood and spleen were examined to facilitate comparison between the peripheral circulation and a principal lymphoid organ. The innate-like lymphocytes express many cell surface antigens but we selected, CXCR3, MHC II, and NKG2D for our phenotypic characterization. Similarly, all three cell types produce a range of cytokines but we selected three mutual mediators to indicate functional activity namely: interferon-gamma (IFNγ), tumor necrosis factor-alpha (TNFα) and perforin.

#### Phenotype

**Figure 5:**
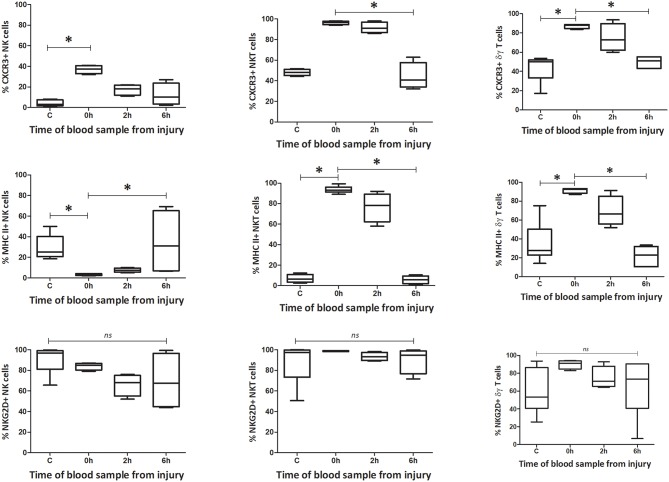
Innate-like cells in blood change their phenotype and function during the first 6 h following T&HS. To determine whether the innate-like cells in this study changed their phenotype after T&HS, flow cytometry was conducted on blood and spleen taken at several sample points during the protocol. This revealed dynamic changes in all three cells, within the first 6 h of injury. Percentage blood phenotype: percentage of CXCR3, MHC II, and NKG2D positive cells. Data are presented as median (min-max) and tested with Kruskal Wallis and Dunns multiple comparison test, *denotes *p* < 0.05.

**Figure 6:**
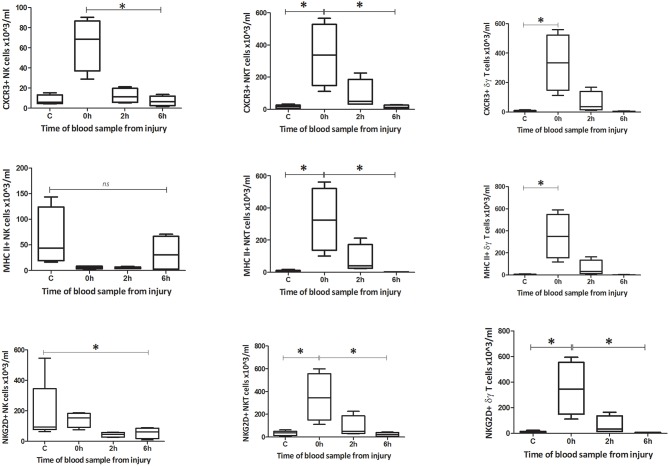
Absolute cell count for blood phenotype: Number of CXCR3, MHC II, and NKG2D positive cells in blood (*n* = 5–8). Data are presented as median (min-max) and tested with Kruskal Wallis and Dunns multiple comparison test, *denotes *p* < 0.05.

**Figure 7:**
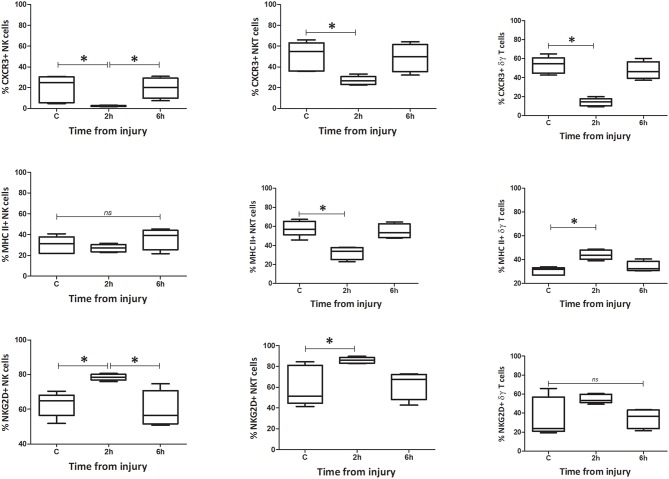
Percentage spleen phenotype: percentage of CXCR3, MHC II, and NKG2D positive cells. Data are presented as median (min-max) and tested with Kruskal Wallis and Dunns multiple comparison test, *denotes *p* < 0.05.

**Figure 8:**
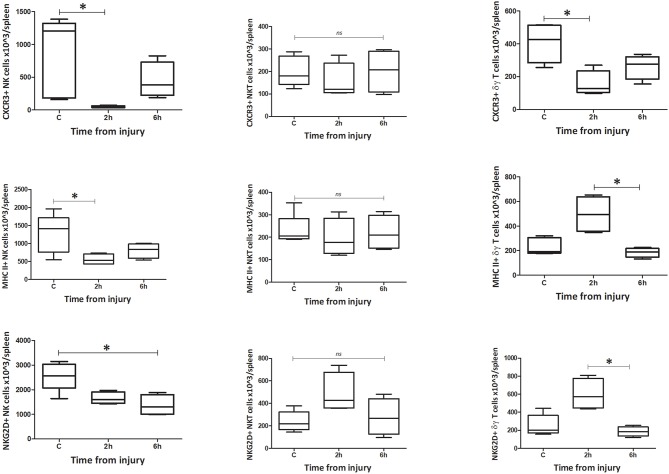
Absolute cell count for spleen phenotype: Number of CXCR3, MHC II, and NKG2D positive cells in spleen (*n* = 5–8). Data are presented as median (min-max) and tested with Kruskal Wallis and Dunns multiple comparison test, *denotes *p* < 0.05.

Within peripheral blood, the percentage and count of CXCR3+ NK1.1+ CD3–, NK1.1+ CD3+, and γδTCR+ CD3+ cells increased immediately following injury (0 h) (Figures 5–8). The percentage and number of CXCR3+ cells then returned to baseline, for all three subsets, by 6 h after injury. By contrast, the percentage and number of CXCR3+ cells within the spleen decreased by 2 h for all three subsets but then also returned to baseline by 6 h. The circulating NK1.1+ CD3– population demonstrated a decrease in MHC II+ cells. This contrasted with a dramatic rise in MHCII+ NK1.1+ CD3+, and γδTCR+ CD3+ cells within blood. Changes in the spleen were less clear-cut, but demonstrated a fall in MHCII+ NK1.1+ CD3– and NK1.1+ CD3+ cells, while MHCII+ γδTCR+ CD3+ cells increased. The number of NKG2D+ NK1.1+ CD3– cells gradually decreased over 6 h, while the number of NKG2D+ NK1.1+ CD3+ and γδTCR+ CD3+ cells increased immediately after T&HS (0 h) and then fell back to baseline. Changes in innate-like cell phenotype with respect to CXCR3, MHCII and NKG2D, were observed at 0 h and 2 h following T&HS. All phenotypic changes had returned to baseline by 6 h (Supplementary Table 2).

#### Function

**Figure 9:**
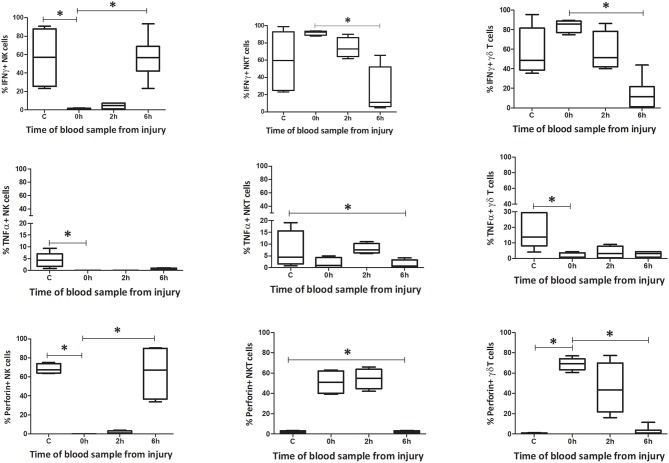
Innate-like cells in the spleen change their phenotype and function during the first 6 h following T&HS. To determine whether the innate-like cells in this study changed their intracellular cytokine levels after T&HS, flow cytometry was conducted on blood and spleen taken at several sample points during the protocol. This revealed extremely dynamic changes in all three cell types, within the first 6 h of injury. Percentage blood: percentage of IFNγ, TNFα, and Perforin positive cells. Data are presented as median (min-max) and tested with Kruskal Wallis and Dunns multiple comparison test, *denotes *p* < 0.05.

**Figure 10:**
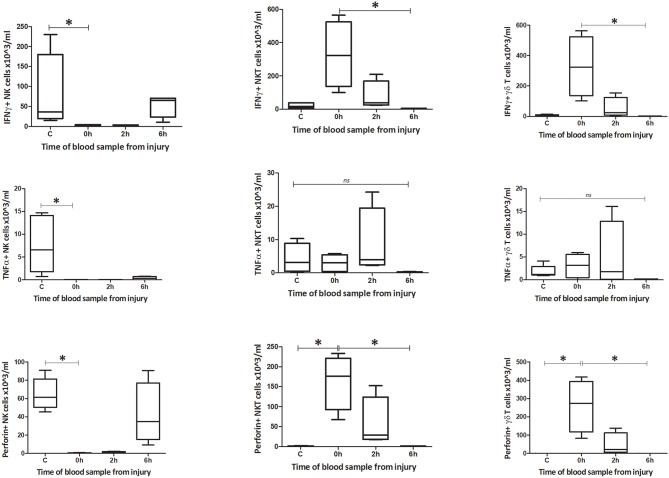
Absolute cell count for blood: number of IFNγ, TNFα, and Perforin positive cells in blood (*n* = 5–8). Data are presented as median (min-max) and tested with Kruskal Wallis and Dunns multiple comparison test, *denotes *p* < 0.05.

**Figure 11:**
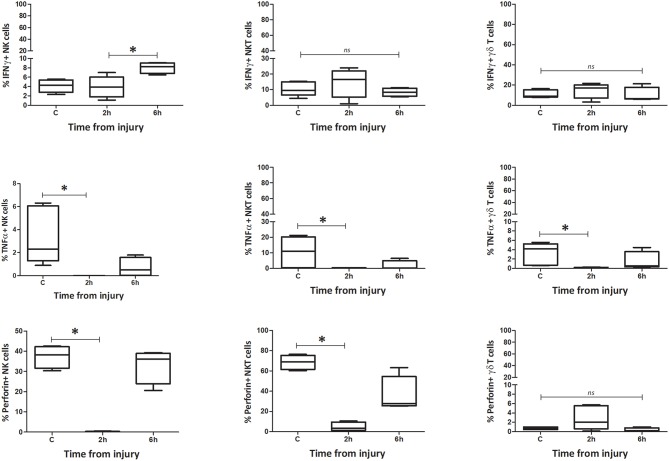
Percentage spleen: percentage of IFNγ, TNFα, and Perforin positive cells. Data are presented as median (min-max) and tested with Kruskal Wallis and Dunns multiple comparison test. *denotes *p* < 0.05.

**Figure 12:**
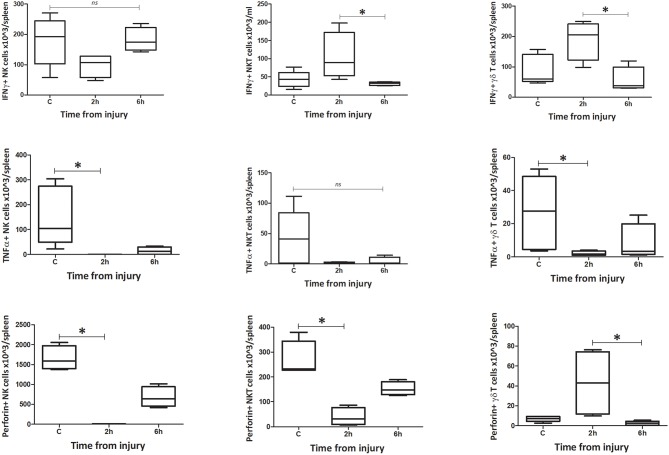
Absolute cell count for spleen: number of IFNγ, TNFα, and Perforin positive cells in spleen (*n* = 5–8). Data are presented as median (min-max) and tested with Kruskal Wallis and Dunns multiple comparison test, *denotes *p* < 0.05.

The number of IFNγ+ NK1.1+ CD3– cells within circulating blood fell precipitously by the 0 h time point, but the population was restored by 6 h (Figures 9–12). Conversely, NK1.1+ CD3+ and γδTCR+ CD3+ cells demonstrated increased intracellular IFNγ at 0 h, that had returned to baseline levels by 6 h. Within the spleen, a slight increase in IFNγ+ NK1.1+ CD3– cells by 6 h was observed, while IFNγ+ NK1.1+ CD3+ and γδTCR+ CD3+ cells displayed a transient rise at 2 h, that returned to baseline by 6 h. Intracellular TNFα was lower in all three cell types within blood and spleen by 2 h following injury. Intracellular perforin levels within the blood borne cells broadly mirrored IFNγ levels. An immediate fall in Perforin+ NK1.1+ CD3– cells was observed, with a gradual increase back to baseline by 6 h. Whereas, NK1.1+ CD3+ and γδTCR+ CD3+ cells demonstrated a sudden increase at 0 h and a reduction back to baseline by 6 h. Within the spleen, a reduction in perforin+ NK1.1+ CD3– and NK1.1+ CD3+ cells was observed at 2 h. Conversely an increase in perforin positive γδTCR+ CD3+ cells was seen at 2 h. Wave-like fluctuations in intracellular IFNγ, TNFα, and perforin were observed in all three innate-like cell types during the 6 h protocol, predominantly with levels returning to baseline by 6 h. T&HS prompted rapid cytokine production by innate-like lymphocytes within blood and spleen (Supplementary Table 2).

### Changes in Phenotype and Function of Innate-Like Lymphocytes Within Lung, Were Comparatively Delayed

**Figure 13:**
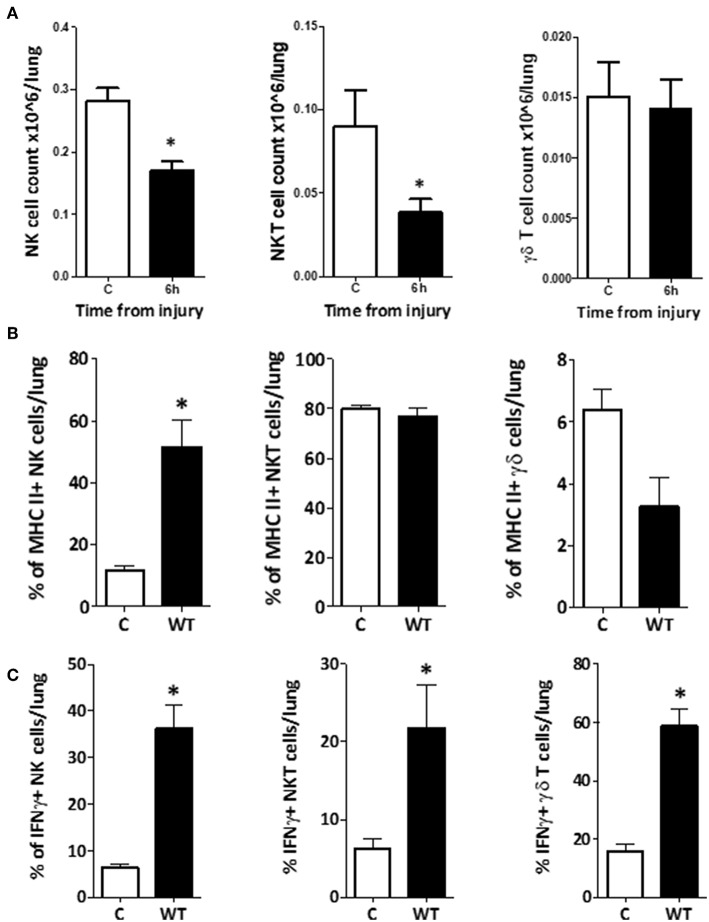
Changes in phenotype and function of innate-like lymphocytes within lung, were comparatively delayed. **(A)** To investigate whether the innate-like lymphocytes within lung also displayed changes in phenotype or function after T&HS, flow cytometry of a single cell suspension of lung tissue was conducted using the same staining protocol. The total number of NK1.1+ CD3– and NK1.1+ CD3+ cells within the lung reduced by 6 h, but no change in γδ TCR+ CD3+ cells was demonstrated. Cell counts (× 10^6^/lung): NK1.1+ CD3–: 0.28 (0.26–0.30) vs. 0.17 (0.15–0.18), *p* = 0.02. NK1.1+ CD3+: 0.09 (0.06–0.11) vs. 0.04 (0.03–0.04), *p* = 0.04. γδ TCR+ CD3+: 0.01 (0.01–0.02) vs. 0.02 (0.01–0.02), *p* = 0.89, *n* = 6–8. Control (C) represents a naïve subject of the same strain and age, *n* = 4–6. Data are presented as mean (SEM) and tested with a Mann Whitney test, *denotes *p* < 0.05. **(B)** NK1.1+ CD3– cells in lung tissue upregulated MHCII expression by 6 h following T&HS. Cell surface phenotype markers on NK1.1+ CD3–, NK1.1+ CD3+, and γδ TCR+ CD3+ cell populations were examined in lung tissue, using flow cytometry at 6 h following injury. The NK1.1+ CD3– cell population demonstrated upregulation of MHC II, while NK1.1+ CD3+ MHCII expression remained the same. γδ TCR+ CD3+ cells appeared to downregulate MHCII, although this did not reach significance. NK1.1+ CD3–: C vs. 6h: 12 (2) vs. 52 (9), *p* = 0.02. NK1.1+ CD3+: C = 80 (2) vs. 77 (4), *p* = 0.56. γδ TCR+ CD3+: C = 6 (1) vs. 3 (1), *p* = 0.09. Control (C) denotes a naïve subject of the same strain and age (*n* = 4–6). Data are presented as median (IQR) and tested with a Mann Whitney test, *denotes *p* < 0.05. **(C)** NK1.1+ CD3–, NK1.1+ CD3+, and γδ TCR+ CD3+ cells within lung tissue, all demonstrated an increase in the % of IFNγ+ cells by 6 h following T&HS. To assess activity of innate-like lymphocytes within lung tissue, intracellular IFNγ, TNFα, and perforin were examined using flow cytometry. At 6 h following T&HS more NK1.1+ CD3–, NK1.1+ CD3+, and γδ TCR+ CD3+ cells within lung tissue, were positive for IFNγ compared with their naïve state. No change in TNFα or perforin production was observed. Control (C) denotes a naïve subject of the same strain and age. C vs. 6 h: NK1.1+ CD3– = 6 (1) vs. 36 (5), *p* = 0.02, NK1.1+ CD3+ = 6 (1) vs. 22 (6), *p* = 0.02, γδ TCR+ CD3+ = 16 (2) vs. 59 (6), *p* = 0.02. *n* = 6–8 and data are presented as median (IQR) and tested with a Mann Whitney test, *denotes *p* < 0.05.

To determine whether the changes in NK1.1+ CD3–, NK1.1+ CD3+, and γδTCR+ CD3+ cells within the lung were consistent with those seen in blood or spleen, we performed the same phenotype and function analyses on the innate-like subsets from lung tissue homogenates. This demonstrated that the populations of NK1.1+ CD3– and NK1.1+ CD3+ cells within lung decreased by 6 h, while the population of γδTCR+ CD3+ cells remained unchanged (Figure 13A). NK1.1+ CD3– cells demonstrated a dramatic increase in the percentage of MHC II+ cells at 6 h following trauma, although no change was observed in NK1.1+ CD3+ or γδTCR+ CD3+ cells (C vs. 6h: NK1.1+ CD3– = 12 (2) vs. 52 (9), *p* = 0.02, NK1.1+ CD3+ = 80 (2) vs. 77 (4), *p* = 0.56, γδTCR+ CD3+ = 6 (1) vs. 3 (1), *p* = 0.09) (Figure 13B). No difference in NKG2D or CXCR3 expression was identified between the C and 6 h timepoint models, in any of the three cell types. All three subsets increased their percentage of IFNγ+ cells by 6 h following T&HS (Figure 13C). No change in TNFα or perforin positive cells was observed [C vs. 6 h: NK1.1+ CD3– = 6 (1) vs. 36 (5), *p* = 0.02, NK1.1+ CD3+ = 6 (1) vs. 22 (6), *p* = 0.02, γδTCR+ CD3+ = 16 (2) vs. 59 (6), *p* = 0.02. *n* = 6–8]. Data are presented as median (IQR) and tested using a Mann-Whitney *U-*test. The responses of the innate like cell subsets in the lung were distinctly different from those seen in blood and spleen. They were characterized by a dramatic rise in MHC II expression on the NK1.1+ CD3– cells and an increase in IFNγ production by all three cell types. In addition, the responses of these lung-based lymphocytes was distinctly delayed when compared to the cells in blood and spleen, where rapid and early changes had predominantly returned to baseline by 6 h.

## Discussion

This study used a well-established murine model of T&HS to define the dynamics of lymphopenia and examine innate-like lymphocyte behavior within different organs, at structured intervals following injury. It confirms that lymphopenia occurs between 2 and 6 h in this murine model, which supports human observations and suggests murine work is of value to this line of investigation (10, 11, 15). Lymphopenia is frequently attributed to widespread apoptosis, but we were unable to demonstrate this (13, 14, 32). We sought evidence of lymphocyte movement out of circulation and into tissues by examining lung, but this was also not significant. There are many other potential destinations for lymphocyte trafficking and examination of injury sites, liver, lymph nodes and gut may be of value in future. Secondly, this study revealed that T&HS provokes an immediate response from the NK1.1+ CD3–, NK1.1+ CD3+, and γδTCR+ CD3+ lymphocyte subsets, with respect to cell count, phenotypic display, and cytokine production. These changes are cell specific, tissue specific and time-dependent, although the responses of NK1.1+ CD3+ and γδTCR+ CD3+ cells often co-aligned. The findings highlight the need for future study examining each cell type individually, with a focus on the hyper-acute phase (<2 h following injury). The immediacy of the response in blood and lymphoid tissues (spleen and bone marrow) is very clear, as 0 h and 2 h time-points reveal the greatest changes and levels frequently returned to baseline by 6 h. By contrast lung tissue responses were still evolving at 6 h, suggesting that the inflammatory response within non-lymphoid organs may lag behind or follow a different course. This study presents novel information about early immune cell behavior following trauma and suggests that in order to determine the contribution of immune cells to MODS development, we may need to examine cells within the organs themselves.

This study has demonstrated that the hyper-acute response in innate-like lymphocytes is complex and dynamic. Further work is required to fully characterize the behavior but certain observations require particular mention. Bone marrow at 6 h demonstrated an increase in total lymphocyte count, which suggests, cell ingress or increased proliferation. Immature cells have been identified in blood after trauma and bone marrow failure has been demonstrated in humans trauma patients with MODS, therefore formal investigation of the hyper-acute bone marrow response may aid understanding of cell production and trafficking and its implication for MODS development (33, 34). In contrast, NK1.1+ CD3– cells within bone marrow had reduced by 6 h. These cells did not remain in circulation and did not accumulate in lung, therefore their destination and role remain unclear. Bone marrow derived hematopoietic progenitor cells are vital to healing at injury sites and so future investigation could examine areas of injury in more detail (35–38). CXCR3 is a migration marker known to recruit NK and NKT cells to sites of injury. This study revealed an immediate (0 h) increase in CXCR3+ populations of blood-borne NK1.1+ CD3– and NK1.1+ CD3+ cells, which supports a potential for migration (33, 39, 40). MHCII expression was increased on circulating NK1.1+ CD3– cells by 6 h, but increased more quickly on circulating NK1.1+ CD3+ and γδTCR+ CD3+ cells. Within lung, MHCII was substantially increased on NK1.1+ CD3– cells at 6 h, while on NK1.1+ CD3+ and γδTCR+ CD3+ cells it remained unchanged. In other disease settings, NK cells are known to acquire MHCII from activated dendritic cells via trogocytosis (41) Activated γδ T cells are also known to acquire MHCII expression and present antigens, but the relevance for NK1.1+ CD3+ cells is less clear (42, 43) As endogenous alarmin molecules are considered the trigger(s) for the sterile inflammatory response to trauma, the presence of MHCII on these innate-like lymphocyte subsets represents a potential mechanism for organ injury and warrants further investigation (44–46). Finally, IFNγ, TNFα, and perforin represent potentially important cytokine mediators during the hyper-acute phase of inflammation. In the blood and spleen populations, cytokine production appeared sequential and orchestrated, with immediate release from NK1.1+ CD3– cells, followed by production and assumed release from NK1.1+ CD3+ and γδTCR+ CD3+. This wave-like signaling was not only extremely rapid but short-lived as concentrations returned to baseline by 6 h, suggesting that other molecules may then be responsible for downstream propagation. These findings suggest that T&HS activate both the pro-inflammatory and cytoxic functions of NK cells and consistent with this notion, we have previously shown that NK1.1+ CD3– cell populations drive the propagation of monocyte infiltration and injury in the liver in this model (35). These results provide a springboard for future research regarding the role of innate-like lymphocytes in the hyperacute inflammatory response to trauma and the pathogensis of MODS.

There are limitations of this study which we acknowledge, primarily the relatively small group sizes. Confirmation of these findings in larger groups and other trauma models would be desirable. More refined NKT cell definition would have been achieved had we included a CD1d marker. Liver necrosis has been demonstrated in this T&HS model which may contribute to the lymphocyte death demonstrated in the spleen at 6 h (47). Further examination of lung tissue at the 0, 2, or 24 h time-points would be required to fully describe the organ events. Investigation of a broader range of immune cells would also be desirable as we have previously demonstrated a key role for innate lymphoid cells in lung injury in this model (48). Lymph node populations and bone marrow cell proliferation were not examined but may be incorporated into future experiments. Cytokine release is assumed but measurement of systemic IFNγ, TNFα, and perforin concentrations at each timepoint would be required for confirmation. Finally, the relevance of these study findings to the development of human MODS is unclear although we hope it adds to the understanding of the hyper-acute inflammatory mechanisms in play following trauma.

In conclusion, this study has demonstrated novel findings regarding the activation and response of three innate-like lymphocyte subsets in the hyper-acute phase of the immunological response to traumatic injury. It demonstrates that the greatest changes in these cells are occurring between 0 and 2 h following injury, reinforcing the importance of early sampling times. It highlights that events within blood are not mirrored by the tissues, therefore study of individual organ tissues will be needed to understand their responses and the pathogenesis of MODS development. These results lend support to the evidence from human studies regarding the potential importance of lymphocytes to the early immune response to injury (10–12, 15). It provides further evidence that the immunological response to trauma is not a dysregulated, dysfunctional reaction, but more likely a consistent, proportional, integrated and complex response (1). Trauma provides a unique population for investigation of these early inflammatory events, as the timing of the initiating stimulus can be precisely determined. Greater understanding of the mechanisms involved in trauma immunology could be extremely valuable, as expediting MODS recovery stands to benefit many other medical specialties.

## Author Contributions

JM devised and conducted all the experiments and wrote the manuscript. RH provided technical support and supervision. SC and MR assisted with data collection. The manuscript was edited by all authors. TB stands as guarantor.

### Conflict of Interest Statement

The authors declare that the research was conducted in the absence of any commercial or financial relationships that could be construed as a potential conflict of interest.
