# Supplementary material for: Innate-Like Lymphocytes Are Immediate Participants in the Hyper-Acute Immune Response to Trauma and Hemorrhagic Shock
Source: Front Immunol. 2019 Jul 11;10:1501. doi: 10.3389/fimmu.2019.01501 (PMC6638190; doi:10.3389/fimmu.2019.01501)
Supplement: Supplementary file 1 [file Table_1.docx]

Table 1: Trauma and haemorrhagic shock initiate dynamic changes in lymphocyte sub-populations which are cell specific, compartment specific and time dependent

|  |  |  |  |  | **p values** | | |
| --- | --- | --- | --- | --- | --- | --- | --- |
|  | **Cell type** | **naïve WT** | **6h** | **24h** | **naïve vs 6h** | **6h vs 24h** | **Kruskal Wallis** |
| ***Blood*** | NK cells | 0.25 (0.21-0.29) | 0.10 (0.08-0.12) | 0.15 (0.14-0.16) | 0.01 | 0.03 | 0.02 |
|  | NK T cells | 0.09 (0.04-0.14) | 0.06 (0.05-0.08) | 0.06 (0.04-0.08) | 0.7 | 0.65 | 0.45 |
|  | γδ T cells | 0.05 (0.04-0.07) | 0.03 (0.02-0.04) | 0.06 (0.04-0.07) | 0.25 | 0.82 | 0.24 |
|  | T helper cells | 0.47 (0.32-0.62) | 0.64 (0.54-0.73) | 0.53 (0.47-0.59) | 0.21 | 0.27 | 0.34 |
|  | Cytotoxic T cells | 0.23 (0.16-0.30) | 0.27 (0.22-0.31) | 0.27 (0.25-0.29) | 0.33 | 0.93 | 0.37 |
|  |  |  |  |  |  |  |  |
| ***Spleen*** | NK cells | 6.45 (5.61-7.29) | 3.57 (3.09-4.05) | 1.98 (1.58-2.38) | 0.02 | <0.01 | <0.01 |
|  | NK T cells | 1.08 (0.88-1.27) | 0.48 (0.36-0.59) | 0.79 (0.70-0.89) | 0.04 | 0.24 | 0.04 |
|  | γδ T cells | 1.47 (1.28-1.66) | 2.85 (2.41-3.29) | 2.56 (2.26-2.86) | 0.01 | 0.01 | 0.02 |
|  | T helper cells | 35.94 (31.19 - 40.69) | 14.83 (11.62 - 18.03) | 18.86 (17.62 - 20.11) | 0.02 | 0.43 | 0.03 |
|  | Cytotoxic T cells | 9.71 (8.29 - 11.12) | 4.99 (4.06 - 5.93) | 7.82 (6.89- 8.76) | 0.03 | 0.25 | 0.04 |
|  |  |  |  |  |  |  |  |
| ***Bone Marrow*** | NK cells | 1.92 (1.83-2.00) | 0.59 (0.56-0.62) | 0.87 (0.76-0.97) | <0.01 | <0.01 | 0.01 |
|  | NK T cells | 0.18 (0.12-0.24) | 0.28 (0.25-0.31) | 0.86 (0.70-1.03) | 0.16 | 0.01 | <0.01 |
|  | γδ T cells | 0.20 (0.16-0.24) | 0.28 (0.23-0.32) | 0.48 (0.44-0.51) | 0.26 | <0.01 | <0.01 |
|  | T helper cells | 0.48(0.43-0.54) | 1.38 (1.17-1.60) | 1.1(0.71-1.52) | 0.02 | 0.18 | 0.04 |
|  | Cytotoxic T cells | 0.47 (0.30 - 0.65) | 1.16 (1.02-1.31) | 1.16 (0.84 - 1.47) | 0.02 | 0.64 | 0.05 |
|  |  |  |  |  |  |  |  |
| Absolute cell counts are expressed as x10^6/ml per ml of blood/per spleen/per both hind limbs | | | | | | | |

Table 2: Innate-like cells within blood and spleen change their phenotype and function during the first 6h following T&HS

|  |  | **Blood** | | | | |  | **Spleen** | | | | |
| --- | --- | --- | --- | --- | --- | --- | --- | --- | --- | --- | --- | --- |
|  |  | **C** | **0h** | **2h** | **6h** | **p value** |  | **C** | **0h** | **2h** | **6h** | **p value** |
| **NK cells (NK1.1+ CD3-)** | **CXCR3** | 3(3-7) | 37(34-40) | 18(14-21) | 10(6-17) | <0.01 |  | 25(7-30) | - | 2(2-3) | 20(14-25) | 0.01 |
|  | **MHC II** | 25(23-31) | 3(3-4) | 8(7-9) | 31(8-57) | <0.01 |  | 31(22-35) | - | 27(24-29) | 39(34-42) | 0.49 |
|  | **NKG2D** | 97(97-99) | 85(83-86) | 68(61-73) | 68(46-91) | 0.13 |  | 65(61-66) | - | 78(78-80) | 56(53-63) | <0.01 |
|  | **IFNγ** | 84(64-90) | 1(0-2) | 5(2-7) | 50(41-54) | <0.01 |  | 4(3-5) | - | 4(3-5) | 8(7-9) | 0.03 |
|  | **TNFα** | 4-(3-5) | 0(0-0) | 0(0-0) | 0(0-1) | <0.01 |  | 2(2-6) | - | 0(0-0) | 1(0-1) | <0.01 |
|  | **Perforin** | 68(65-71) | 0(0-0) | 1(1-2) | 46(34-89) | <0.01 |  | 38(34-42) | - | 0-(0-0) | 36(31-38) | 0.01 |
| **NKT cells (NK1.1+ CD3+)** | **CXCR3** | 47(44-49) | 96(95-97) | 91(88-94) | 41(37-48) | <0.01 |  | 55(36-60) | - | 27(24-28) | 50(42-57) | 0.02 |
|  | **MHC II** | 7(5-8) | 93(93-93) | 78(71-83) | 6(4-8) | <0.01 |  | 57(56-63) | - | 34(27-38) | 53(49-59) | 0.01 |
|  | **NKG2D** | 97(96-99) | 99(99-99) | 93(90-96) | 95(88-97) | 0.28 |  | 51(48-78) | - | 86(83-88) | 68(59-71) | 0.02 |
|  | **IFNγ** | 60(27-87) | 92(91-93) | 73(69-79) | 11(10-25) | 0.03 |  | 10(9-15) | - | 16(10-20) | 8(7-10) | 0.43 |
|  | **TNFα** | 4(3-8) | 1(0-2) | 8(7-9) | 1(0-2) | 0.04 |  | 11(0-19) | - | 0(0-0) | 0(0-2) | 0.02 |
|  | **Perforin** | 2(1-2) | 51(42-60) | 55(50-60) | 2(2-3) | <0.01 |  | 69(64-73) | - | 3(2-8) | 28(26-37) | <0.01 |
| **γδ T cells (γδ TCR+ CD3+)** | **CXCR3** | 50(49-51) | 88(87-89) | 73(66-81) | 52(50-55) | <0.01 |  | 55(47-57) | - | 15(11-15) | 46(44-50) | <0.01 |
|  | **MHC II** | 26(23-45) | 93(91-93) | 67(63-73) | 18(11-30) | <0.01 |  | 32(27-32) | - | 44(42-47) | 32(32-35) | 0.01 |
|  | **NKG2D** | 59(43-82) | 91(88-93) | 71(67-78) | 71(47-86) | 0.14 |  | 24(22-48) | - | 53(53-59) | 36(29-42) | 0.06 |
|  | **IFNγ** | 58(45-75) | 86(82-88) | 51(46-62) | 11(3-19) | <0.01 |  | 9(8-14) | - | 17(11-19) | 7(6-11) | 0.42 |
|  | **TNFα** | 19(10-28) | 1(0-2) | 3(1-6) | 2(1-4) | <0.01 |  | 4(1-5) | - | 0(0-0) | 1(0-2) | <0.01 |
|  | **Perforin** | 1(1-1) | 69(66-71) | 44(33-55) | 1(0-3) | <0.01 |  | 1(0-1) | - | 2(1-5) | 0(0-0) | 0.09 |
| Data are presented as % of positive cells | | | | | | | | | | | | |
